# Diagnostic performance of panfungal PCR on tissue specimens for the diagnosis of invasive fungal diseases: a systematic review and meta-analysis of the Fungal PCR Initiative (FPCRI)

**DOI:** 10.1128/jcm.00721-26

**Published:** 2026-08-21

**Authors:** Paraskevas Filippidis, Céline El Khoury, Mario Cruciani, Alexandre Alanio, Rosemary A. Barnes, Ralf Bialek, Maria Jose Buitrago, J. Peter Donnelly, Raphael Grolimund, Ferry Hagen, Catriona Halliday, Elizabeth Johnson, Martina Lengerova, Jurgen Loeffler, Willem J. G. Melchers, Carlo Mengoli, Lily Novak-Frazer, Onya Opota, Melinda Paholcsek, Thomas F. Patterson, Riina Rautemaa-Richardson, Volker Rickerts, Markus Ruhnke, Lena Tschiderer, Alexia Cavin-Trombert, Birgit Willinger, P. Lewis White, Frederic Lamoth

**Affiliations:** 1 Service of Infectious Diseases, Department of Internal Medicine, Lausanne University Hospital and University of Lausanne 569549, Lausanne, Switzerland; 2 The Fungal PCR Initiative, a working group of the International Society of Human and Animal Mycology, Verona, Italy; 3 Laboratory of Mycology-Parasitology, Saint-Louis Hospital 55663 , Paris, France; 4 Translational Mycology Research Group, Mycology Department, Institut Pasteur, Université Paris Cité, National Reference Centre for Invasive Mycoses and Antifungals 555089, Paris, France; 5 Department of Infection, University of Cardiff 2112, Cardiff, United Kingdom; 6 LADR GmbH Medizinisches Versorgungszentrum Dr. Kramer & Kollegen 234569 , Geesthacht, Germany; 7 Reference Mycology Laboratory, National Centre for Microbiology, Instituto de Salud Carlos III 38176, Madrid, Spain; 8 CIBERINFEC, ISCIII-CIBER de Enfermedades Infecciosas, Instituto de Salud Carlos III, Madrid, Spain; 9 The European PCR Initiative Foundation, Nijmegen, the Netherlands; 10 Medical Library, Lausanne University Hospital and University of Lausanne 30635, Lausanne, Switzerland; 11 Department of Medical Microbiology, University Medical Center Utrecht, Utrecht, the Netherlands; 12 Centre for Infectious Diseases and Microbiology Laboratory Services, NSW Health Pathology, Westmead Hospital 441551, Westmead, NSW, Australia; 13 National Mycology Reference Laboratory, UK Health Security Agency, Bristol, United Kingdom; 14 Department of Laboratory Medicine, University Hospital Brno, Brno, Czech Republic; 15 Universitaetsklinikum Wuerzburg, Medizinische Klinik II, Wuerzburg, Germany; 16 Department of Medical Microbiology, Radboud University Medical Center 6034, Nijmegen, the Netherlands; 17 The Fungal PCR Initiative, a working group of the International Society of Human and Animal Mycology, Padua, Italy; 18 NHS Mycology Reference Centre Manchester (MRCM), University of Manchester 5292, Manchester, United Kingdom; 19 Institute of Microbiology, Lausanne University Hospital and University of Lausanne 536517, Lausanne, Switzerland; 20 Faculty of Agricultural and Food Sciences and Environmental Management, University of Debrecen, Complex Systems and Microbiome-innovations Centre 72292, Debrecen, Hungary; 21 University of Texas Health San Antonio 14742, San Antonio, Texas, USA; 22 Mycology Reference Centre Manchester and Department of Infectious Diseases, Wythenshawe Hospital, Manchester University NHS Foundation Trust 5293, Manchester, United Kingdom; 23 Division of Evolution, Infection and Genomics, Faculty of Biology, Medicine and Health, University of Manchester 12203, Manchester, United Kingdom; 24 Robert Koch Institute 9222, Berlin, Germany; 25 Department of Hematology, Oncology and Palliative Care, Helios Klinikum Aue 62468, Aue, Germany; 26 Institute of Clinical Epidemiology, Public Health, Health Economics, Medical Statistics and Informatics, Medical University of Innsbruck, Innsbruck, Austria; 27 Division of Clinical Microbiology, Department of Laboratory Medicine and Comprehensive Center for Infection Medicine, Medical University of Vienna 27271, Vienna, Austria; 28 Public Health Wales Mycology Reference Laboratory, University Hospital of Wales 97609 , Cardiff, United Kingdom; 29 Division of Infection and Immunity, School of Medicine, Cardiff University, Cardiff, United Kingdom; University of Utah Health Medicine, Salt Lake City, Utah, USA

**Keywords:** fungal PCR, aspergillosis, mucormycosis, mycoses, biopsy

## Abstract

Invasive fungal diseases are difficult to diagnose because of the limited sensitivity of culture. Panfungal PCR amplicon sequencing assays (targeting ribosomal RNA, such as 18S, 28S, ITS) are recommended for fungal identification in histopathology samples showing fungal elements. However, data describing its overall performance and consistency are lacking. This systematic literature review and meta-analysis assessed the performance of panfungal PCR on formalin-fixed paraffin-embedded (FFPE) and non-fixed (fresh or frozen) tissue samples. A systematic literature search was performed to include studies reporting the use of panfungal PCR for fungal identification in FFPE or non-fixed tissue samples. PCR sensitivity and specificity were assessed using the reference standard of histopathology showing fungal elements. Quality assessment was performed using the Quality Assessment of Diagnostic Accuracy Studies (QUADAS-2) tool. Pooled estimates were obtained using random-effects meta-analysis. Twenty-eight studies were included. In FFPE samples (18 studies, 852 samples), sensitivity and specificity were 75.4% (95% confidence interval [CI], 59.2–86.6) and 93.5% (70.2–98.9), respectively. Sensitivity in non-fixed samples (13 studies, 207 samples) was 86.5% (74.7–93.3), while specificity could not be assessed (insufficient data). Comparative analyses showed a significantly higher sensitivity of panfungal PCR over culture (88.2%; 76–94.7 vs 52.2%; 39–65, *P* = 0.001). Sub-analyses could not demonstrate the superiority of one PCR target over another due to limited data. Panfungal PCR exhibited adequate sensitivity and good specificity in FFPE samples. Sensitivity was even higher in non-fixed samples and largely superior to culture. Nevertheless, large interstudy variability was observed, warranting interlaboratory studies to define the optimal PCR target and standardized protocols.

**IMPORTANCE:**

Invasive fungal diseases are difficult to diagnose because of the low sensitivity of culture. Panfungal PCRs are widely used for fungal identification in tissue specimens but suffer from heterogeneous procedures and performance. This meta-analysis shows an acceptable sensitivity (75.4% and 86.5% in fixed and non-fixed samples, respectively) and good specificity (93.5%) of panfungal PCR, supporting its use, not only on histopathology-positive fixed samples but also in non-fixed samples concomitantly with other diagnostic tools (cultures and fungal-specific PCRs if available). These results provide a strong basis for further standardization of panfungal PCR techniques via interlaboratory assays to assess reproducibility and optimize analytical protocols.

**CLINICAL TRIALS:**

This study is registered with PROSPERO as CRD42023461148.

## INTRODUCTION

Invasive fungal diseases (IFD) have a high impact on morbidity and mortality in the increasing population of patients with immunosuppressive conditions (1, 2). The diagnosis of IFD is challenging because of the low sensitivity of culture and the frequent need for invasive procedures to obtain deep tissue samples (3). Demonstration of fungal elements in normally sterile tissues provides a diagnosis of proven IFD (4). However, in many cases (particularly when antifungal therapy is ongoing), culture remains sterile and identification of the fungal pathogen is not possible, which can complicate antifungal therapy. Molecular diagnostic tools (i.e., polymerase chain reaction, PCR), including panfungal PCR (targeting conserved ribosomal RNA regions, such as 18S, 28S, or ITS regions) or genus/species-specific PCRs, have improved the sensitivity and accuracy of IFD diagnosis (5, 6). Standardized fungal PCR protocols and commercial kits have been developed for some specific fungi (e.g., *Aspergillus* spp., *Mucorales*) (7–9). Several studies assessed the performance of *Aspergillu*s-specific or *Mucorales*-specific PCRs in biological fluids (e.g., serum, plasma, and bronchoalveolar lavage fluid) (10, 11). A meta-analysis also provided data on the performance of *Mucorales* PCR in tissue samples (10). Moreover, *Aspergillus* PCR has now been implemented as a mycological criterion in the revised definitions of IFD by the European Organization for Research and Treatment of Cancer (EORTC) and Mycoses Study Group Education and Research Consortium (MSGERC) (4). This group also recommended performing panfungal PCR with amplicon sequencing when fungal elements are seen at histopathological examination of formalin-fixed paraffin-embedded (FFPE) tissue samples (4, 12). However, the sensitivity and specificity of this approach remain uncertain, as fixed samples contain degraded DNA and are prone to contamination, leading to a risk of false-negative and false-positive results, respectively. Systematic analyses or interlaboratory studies addressing panfungal PCRs are currently lacking. Moreover, the relevance of testing fresh tissue samples should be investigated.

The aim of this study was to assess the performance of panfungal PCR in FFPE and non-fixed (fresh or frozen) tissue samples for fungal pathogen identification in IFD.

## MATERIALS AND METHODS

### Study design and search strategy

This systematic review and meta-analysis followed the Preferred Reporting Items for Systematic Review and Meta-Analysis (PRISMA) guidelines for reporting and the PRISMA Statement for Reporting Literature Searches in Systematic Reviews (PRISMA-S) (Material S1) (13, 14). The protocol was registered in PROSPERO (CRD42023461148).

The selected information sources were Medline Ovid ALL, Embase.com, Cochrane Library Wiley (Cochrane Database of Systematic Reviews and Cochrane CENTRAL), Web of Science Core Collection, and Google Scholar.

The search strategies were developed in collaboration with a health information specialist. An initial search strategy was designed for Embase.com, combining index and free-text terms describing the concepts of IFD, PCR, sterile specimen, and standard methods. The preliminary strategy was then translated for the remaining information sources. The final search strategies (detailed in Material S2) were peer-reviewed by another health information specialist using the PRESS checklist (15). No date or language limits were applied. Conference abstracts and conference reviews were filtered out based on publication type. The searches were run on 3 May 2023 and updated on 27 May 2025. A complementary backward and forward citation search was run on 7 April 2025 and updated on 7 August 2025 using citationchaser (https://estech.shinyapps.io/citationchaser/). This tool was eventually used instead of Web of Science Core Collection as it allows users to search for citations and references from multiple publications at once.

All references were imported into EndNote20 (Clarivate, Philadelphia, PA), and duplicates were removed using Deduklick’s automated deduplication algorithm (https://www.risklick.ch/deduklick). Remaining articles were imported into Rayyan (https://www.rayyan.ai) for screening. The results of the citation search were manually deduplicated against the results previously screened.

### Eligibility criteria

Studies were eligible if they reported results of panfungal PCR in human tissue specimens from normally sterile sites for the diagnosis of IFD concomitantly with histopathology results of samples from the same tissue specimen (for FFPE samples) or a companion tissue specimen (for non-fixed samples). The minimal sample size was ≥5 samples per study. Studies comprising a mix of normally sterile and potentially non-sterile tissues (e.g., skin, mucosa) were included if sterile tissues represented ≥80% of samples. Studies reporting panfungal PCR results on rhino-sinusal samples of patients with rhino-sino-orbital mucormycosis were included, considering that recovery of *Mucorales* in such samples was true infection (not potential contamination). Studies comprising a mix of sterile tissues and biological fluids were included if tissue samples represented ≥80% of the data set (pooled data) or when they could be distinguished from fluid samples. Eligible assays comprised panfungal PCR targeting the ribosomal RNA gene complex (18S rRNA gene, 28S rRNA gene, and/or ITS regions).

Exclusion criteria were as follows: (i) studies focusing on endemic mycoses (e.g., histoplasmosis), cryptococcosis, or pneumocystosis; (ii) studies reporting only use of multiplex PCR panels or specific-fungal PCRs; (iii) studies with a data set <5 samples for each type of sample (FFPE or non-fixed); (iv) studies in which non-sterile tissue samples represented >20% of the data set; (v) studies in which biological fluids could not be distinguished from tissue samples or represented >20% of a pooled data set; (vi) studies in which animal tissue could not be distinguished from human tissue samples or represented >20% of a pooled data set; (vii) studies in which results from FFPE and non-fixed tissue samples could not be distinguished; (viii) studies with PCR results provided as positive or negative without further identification by sequencing. Studies reporting results from research laboratories or validation cohorts were not excluded.

### Study selection and data extraction

Titles and abstracts were screened independently by two authors, and full texts were assessed for eligibility by three authors. Potential discrepancies were discussed by a consortium of three authors. Data extraction was performed using a standardized reporting form. Data were collected regarding tissue type (FFPE or non-fixed), specimen type (biopsy or autopsy), tissue origin (lung or non-lung), PCR locus target (18S, 28S, and/or ITS), PCR type (conventional or real-time), histopathology results, concomitant culture results (if available), results of panfungal PCR and of specific-fungal PCRs (e.g., *Aspergillus* spp., *Mucorales*, if available).

### Quality assessment

Methodological quality, including risk of bias and applicability, was assessed independently by two authors using the Standards for Reporting of Diagnostic Accuracy and the Quality Assessment of Diagnostic Accuracy Studies (QUADAS-2) tools (16–18). Disagreements were resolved by a third author blinded to the previous reviews. QUADAS-2 consists of the following four key domains: patient selection, index test, reference standard, flow and timing. Each item is assessed in terms of risk of bias and the first three in terms of concerns regarding applicability. Signaling questions are included to assist in judgments about risk of bias. Risk of bias is judged as “low,” “high,” or “unclear.” If all signaling questions for a domain are answered “yes,” then risk of bias can be judged as “low.” If any signaling question is answered “no,” this flags the potential for bias. The “unclear” category is used only when insufficient data are reported to permit a judgment.

### Data analyses

PCR sensitivity was assessed for FFPE and non-fixed tissue samples using the reference standard of positive histopathology for fungus. For studies reporting results of different PCR targets, the optimal sensitivity was used for the global meta-analysis. When available, individual results of the different targets were treated separately in the sub-analysis comparing their performance.

PCR results were considered true positive if the fungal pathogen was identified at least at genus level and was consistent with the histopathological morphology of the fungus (i.e., “yeast,” “thin, septate,” “broad, non-septate,” or “unspecified” hyphae). False negative results were considered in case of negative PCR results or positive PCR amplification with either failure of sequencing (i.e., no identification or mixed sequences) or identification of a non-pathogenic fungus (or other non-pathogenic non-fungal microorganism, such as plants or algae) considered as contamination or a pathogenic fungus inconsistent with histopathology results. Specificity was assessed if samples with negative histopathology and no IFD diagnosis by an alternative method (e.g., culture) have been tested. True negative results were considered in the absence of PCR amplification. False positive results were considered in case of positive PCR for any fungus.

Comparative analyses were performed to assess the sensitivity of panfungal PCR versus culture and versus fungal-specific PCRs (*Aspergillus* or *Mucorales*) in studies providing front-to-front comparisons.

### Statistical methods

Meta-analyses of sensitivity and specificity were performed using the R package *meta* v8.2-0. Study-level sensitivities were logit-transformed and modeled with a random-effects generalized linear mixed model (GLMM) with logit link, which directly accounts for the binomial distribution and avoids continuity corrections for studies reporting perfect sensitivity and specificity (19). Confidence intervals for individual studies were calculated with the Clopper–Pearson exact method. Between-study variance (τ²) was estimated by restricted maximum likelihood (20, 21). Heterogeneity was assessed using the Q statistic and its *P* value and quantified with the *I*² statistic and its 95% CI (22, 23). Subgroup analyses, including tissue origin (biopsy vs autopsy), PCR target (28S, 18S, ITS), PCR protocol (real-time vs conventional PCR), and use of human DNA extraction controls (for FFPE samples), were conducted using mixed-effects meta-regression analysis by including these variables as fixed effects within the random-effects GLMM to explore sources of heterogeneity (R package *metafor*). Comparisons of histopathology versus culture and panfungal versus fungal-specific PCRs were conducted using mixed-effects meta-regression to account for paired within-study comparisons (R package *metafor*). Bivariate random-effects models were planned to jointly estimate sensitivity and specificity. All analyses were performed using R v4.5.1.

## RESULTS

**Fig 1:**
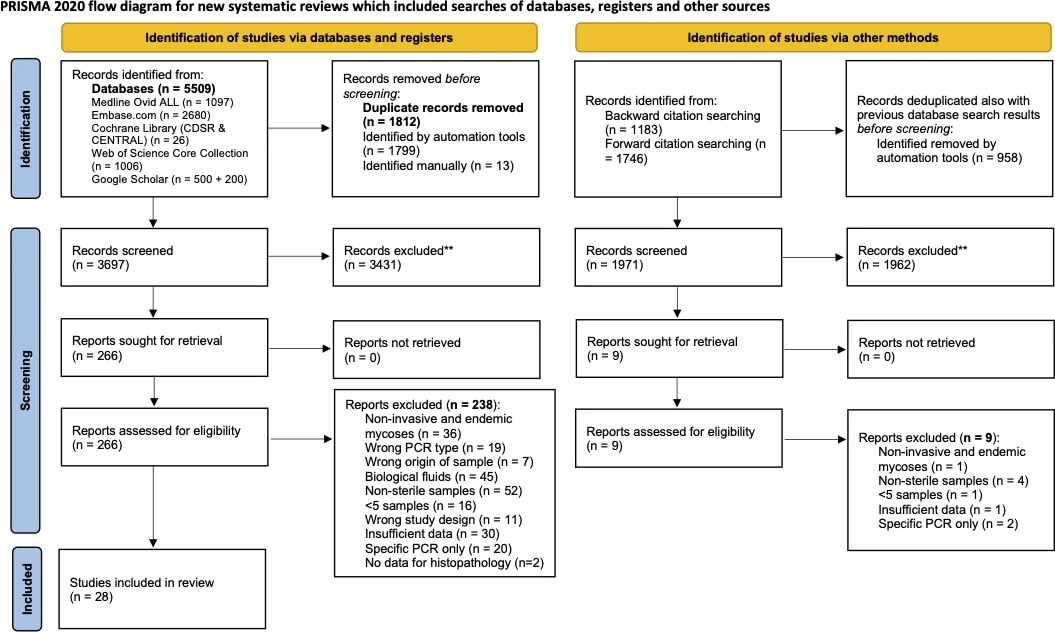
Literature search flowchart. PRISMA flow diagram of literature search and study selection process. The number of records identified, screened, excluded, and included is shown according to PRISMA guidelines. **Records excluded manually by human reviewers.

**Fig 2:**
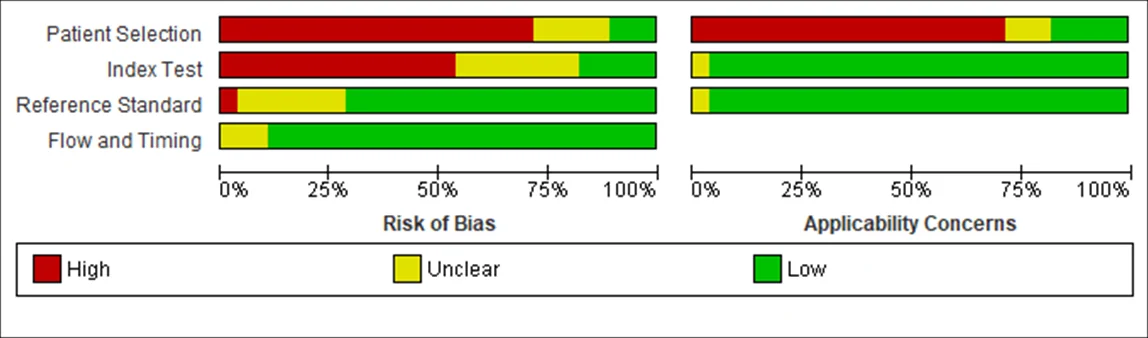
Risk of bias and applicability concerns. Global assessment of risk of bias and applicability concerns graph: review authors’ judgments about each domain presented as percentages across included studies. The methodological quality of the included studies is evaluated using the Quality Assessment of Diagnostic Accuracy Studies (QUADAS-2) tool. Patient selection: risk of bias and applicability concerns related to the method of patient selection (consecutive or random selection? Case-control design avoided? Occurrence of inappropriate inclusions?). Index test: risk of bias and applicability concerns related to the procedure and interpretation of the index test (interpretation without knowledge of the reference standard results? Pre-specified threshold? Procedure and interpretation of the index test correspond to the review question?). Reference standard: risk of bias and applicability concerns related to the procedure and interpretation of the reference standard (reference standard likely to correctly classify the target condition? Reference standard results interpreted without knowledge of the index test results? Target condition as defined by the reference standard corresponds to the review question?). Flow and timing: risk of bias related to the patient flow (appropriate time interval between the index test and reference standard? Same reference standard for all patients? All patients included in the analysis?).

The selection process is shown in a PRISMA flow diagram (Fig. 1). A total of 5,509 records were identified through database searching. After removing 1,812 duplicates, 3,697 unique records were screened for title and abstract, of which 266 were assessed on their full text. Twenty-eight studies met the inclusion criteria. The citation searches yielded 1,971 unique references, of which 9 were assessed on their full text, although none were included. In total, 28 studies were included (15 with FFPE samples only, 10 with non-fixed samples only, and 3 with both types of samples that could be distinguished). The characteristics of these studies are shown in Table 1. Most studies reported panfungal PCR results for all IFD types and included a mix of different sample types, except for three studies, including only cases of invasive mucormycosis and one study with a majority (>90%) of lung tissue samples. Most studies (24/28) reported use of a panfungal PCR targeting the ITS region (alone, *n* = 17 or combined with 28S or 18S, *n* = 7). In three studies, results were reported separately for different PCR targets (Table 1). Individual results for 28S and 18S targets could be obtained in five and two studies, respectively. Results of quality assessment are shown in Fig. 2 (with details of each individual study in Material S3). While the risk of bias of included studies was acceptable, there were substantial concerns about applicability relating to patient selection.

**TABLE 1:** Characteristics of included studies^*a*^

Study (first author)^*b*^	Specimen type	Sample origin	Tissue type^*c*^	IFD type	Available culture results	Fungal-specific PCR results	PCR target^*d*^	Reference
Ala Houhala	Non-fixed	NA	Non-lung (maj)	All	Yes	No	28S	(24)
Babouee 2013	Non-fixed	NA	Lung (maj)	All	Yes	No	ITS	(25)
Babouee Flury 2014	FFPE	NA	Lung (maj)	All	Yes	No	ITS	(26)
Buitrago	FFPE	Biopsy	Lung (maj)	All	Yes	No	ITS	(27)
Clark	FFPE	NA	NA	All	No	No	ITS	(28)
Gade	FFPE	Mixed	Non-lung (maj)	All	No	No	ITS, 28S	(29)
Gomez	FFPE, non-fixed	Biopsy	NA	All	No	No	ITS-28S	(30)
Hendolin	Non-fixed	Mixed	Non-lung (maj)	All	Yes	No	ITS	(31)
Imhof	Non-fixed	Biopsy	Non-lung (maj)	All	Yes	No	18S	(32)
Jillwin	FFPE	Mixed	NA	All	No	Yes (*Mucorales*)	ITS	(33)
Jung	FFPE	NA	NA	All	No	No	ITS	(34)
Larkin	FFPE	Mixed	Non-lung (maj)	All	No	No	ITS	(35)
Lau	FFPE, non-fixed	NA	Non-lung (maj)	All	Yes	No	ITS	(36)
Lysen	FFPE	NA	NA	All	No	No	ITS-28S	(37)
Munoz-Cadavid	FFPE	NA	Non-lung (maj)	All	No	No	ITS	(38)
Rampini	Non-fixed	Biopsy	Non-lung	All	Yes	No	ITS	(39)
Rickerts 2011	FFPE	Mixed	Lung (maj)	All	No	No	ITS-28S	(40)
Rickerts 2012	FFPE	NA	Lung (maj)	All	No	No	ITS, 28S	(41)
Risum	Non-fixed	Biopsy	Non-lung (maj)	IM only	Yes	Yes (*Mucorales*)	ITS, 18S	(42)
Sabino	Non-fixed	NA	Non-lung	All	Yes	No	ITS	(43)
Shinozaki	FFPE	Autopsy	Lung (maj)	All	No	Yes (*Aspergillus*)	ITS	(44)
Sparks	FFPE	Biopsy	NA	All	No	No	ITS	(45)
Springer 2016	Non-fixed	NA	Lung (maj)	IM only	Yes	Yes (*Mucorales*)	ITS	(46)
Springer 2019	FFPE	NA	NA	All	No	Yes (*Aspergillus*)^*e*^	28S	(47)
Stempak	Non-fixed	Biopsy	Non-lung	All	Yes	No	ITS-18S	(48)
Wilmes	FFPE	Biopsy	Non-lung (maj)	All	No	No	28S	(49)
Zaman	Non-fixed	Biopsy	Non-lung	IM only	Yes	Yes (*Mucorales*)	ITS	(50)
Zeller	FFPE, non-fixed	NA	Non-lung (maj)	All	Yes	No	ITS	(51)

^*a*^ FFPE, formalin-fixed paraffin-embedded; NA, information not available; IFD, invasive fungal disease; IM, invasive mucormycosis; ITS, internal transcribed spacer region.

^*b*^ Studies from the same first author are distinguished by year of publication.

^*c*^ Lung, exclusively lung samples; lung (maj), majority (>60%) of lung samples; non-lung, exclusively non-lung samples; non-lung (maj), majority of non-lung samples (>60%).

^*d*^ PCR targets are separated by a comma (,) when results have been provided separately or by a dash (-) when results have been pooled.

^*e*^ Data on Mucorales-PCR were available for only two histopathology-positive samples.

### Performance of panfungal PCR in FFPE tissue samples

**Fig 3:**
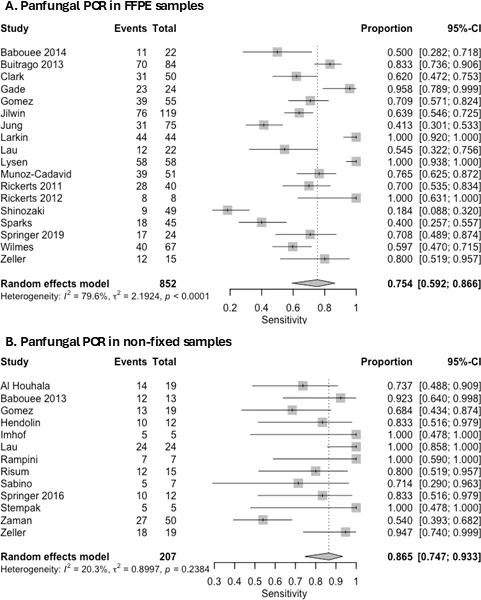
Sensitivity of panfungal PCR for fungal detection/identification in histopathology-positive tissue samples. Forest plots of sensitivity of panfungal PCR in (**A**) FFPE and (**B**) non-fixed tissue samples compared to histopathology (reference). Sensitivity estimates are presented with 95% confidence intervals. Pooled estimates were obtained using a random-effects model. Study heterogeneity is represented by the *I*^2^ statistic.

For the analysis of FFPE samples, 18 studies comprising 852 samples were included (26–30, 33–38, 40, 41, 44, 45, 47, 49, 51). Use of PCR extraction controls for human DNA was reported in 10 (56%) of them (26, 30, 33, 38, 40, 41, 44, 45, 47, 49). Panfungal PCR demonstrated a pooled sensitivity of 75.4% (95% confidence interval [CI], 59.2–86.6) with substantial heterogeneity (*I*² = 79.6%; *P* < 0.0001) (Fig. 3A). To explore sources of heterogeneity, meta-regression analyses were performed and showed no significant differences according to tissue type (biopsy vs autopsy), tissue origin (predominantly [>60%] lung vs non-lung tissue samples), PCR target (28S, 18S, ITS), PCR protocols (real-time vs conventional), and use (or not) of human DNA extraction controls (Material S4). Specificity was 93.5% (70.2–98.9) with high heterogeneity (*I*² = 80.9%; *P* < 0.001) across five studies (280 samples) with appropriate negative controls (Material S5) (28, 30, 45, 47, 51). A bivariate model to analyze the correlation between sensitivity and specificity did not converge owing to the limited number of studies reporting both outcomes.

### Performance of panfungal PCR in non-fixed tissue samples

For the analysis of non-fixed tissue samples, the data set consisted of 13 studies and a total of 207 samples (24, 25, 30–32, 36, 39, 42, 43, 46, 48, 50, 51). Using histopathology as the reference standard, sensitivity was 86.5% (74.7–93.3; *N* = 13) with low heterogeneity (*I*² = 20.3%; *P* = 0.238) (Fig. 3B). Meta-regression showed no significant effect in subgroup analyses (Material S4). Only two studies provided individual sampling data for specificity calculation (*n* = 20 samples) (30, 51), which was insufficient for the meta-analysis.

### Comparative performance of panfungal PCR versus culture

**Fig 4:**
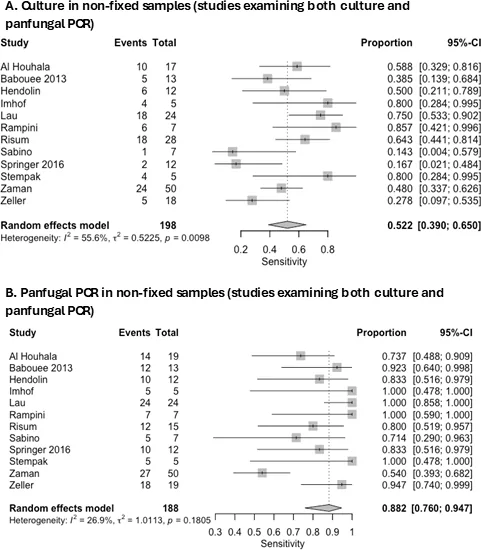
Comparative analysis of sensitivity between culture and panfungal PCR for fungal detection/identification in histopathology-positive tissue samples. Forest plots comparing sensitivity of (**A**) culture versus (**B**) panfungal PCR in non-fixed tissue samples from studies examining both assays. Sensitivity estimates are presented with 95% confidence intervals. Pooled estimates were obtained using a random-effects model. Study heterogeneity is represented by the I^2^ statistic.

This analysis was restricted to non-fixed tissue samples, for which concomitant culture results were available. In 12 comparative studies, the sensitivity of panfungal PCR was significantly higher compared to that of culture (88.2%; 76–94.7 vs 52.2%; 39–65, *P* < 0.001, Fig. 4). Culture results showed moderate heterogeneity (*I*² = 55.6%; *P* = 0.01), in contrast to low heterogeneity for PCR (*I*² = 26.9%; *P* = 0.18).

### Comparative analysis of panfungal PCR versus fungal-specific PCRs

Studies comparing results of panfungal PCR with *Aspergillus*-specific or *Mucorales*-specific PCRs on the same samples were included in this analysis. Pooled data of three studies (all using non-fixed tissue samples) showed that there was a trend toward higher sensitivity of *Mucorales* PCR compared to panfungal PCR (94.8%, 61.0–99.5 vs 69.7%, 48.3–85.0, *P* = 0.1, Material S6). Comparison with *Aspergillus* PCR was not feasible because of limited data (only two studies; *N* = 64 samples) (44, 47).

## DISCUSSION

This meta-analysis of the performance of panfungal PCR for fungal identification in histopathology-positive samples showed an overall sensitivity of 75.4% and 86.5% in FFPE and non-fixed tissue samples, respectively. Failure of PCR to identify the fungus may have resulted from insufficient volume of remaining tissue sample, lack of free DNA amplification (DNA degradation or altered quality of FFPE samples), PCR inhibition, failure of sequencing, or results deemed as exogenous contamination. The use of human DNA extraction controls was not associated with improved sensitivity in our meta-regression analysis. However, these results should be interpreted cautiously because of the small sample size, the lack of details about extraction procedures, and the absence of quantitative results of extracted human DNA.

Heterogeneity and lack of detailed protocols did not allow further investigation of the impact of internal and external control procedures on PCR performance.

False interpretation (i.e., PCR result inconsistent with histopathology result) may also result from inaccurate presumptive histopathologic identification (i.e., *Mucorales* vs *Aspergillus* or other septate mold), for which diagnostic accuracy does not exceed 75%–80% (52, 53), or from co-infections (mainly invasive aspergillosis and mucormycosis), which may occur in a substantial proportion of cases (47, 54, 55). Whether the origin of the tissue sample could affect PCR sensitivity is unclear. Despite a large heterogeneity of tissue samples, we tried to distinguish studies, including predominantly lung or non-lung tissue samples. Although we observed a trend toward increased sensitivity in non-lung compared to lung tissue samples (85% vs 67%, respectively, Material S4), this difference was not statistically significant.

The comparative sensitivity with culture could not be assessed for FFPE samples, for which concomitant culture results were not available in most studies. For non-fixed samples, our results indicated that the sensitivity of PCR was better (88.2% vs 52.2% for culture). While culture is a standard diagnostic approach to obtain fungal identification and antifungal susceptibility profile, PCR testing could be performed concomitantly in fresh samples to improve sensitivity if sufficient material is available. This approach may be particularly useful for patients already receiving antifungal therapy, which can drastically decrease culture sensitivity.

We also compared the performance of panfungal PCR with that of fungal-specific PCRs. This analysis was possible for *Mucorales* PCR only. While *Mucorales* PCR tended to have better sensitivity compared to panfungal PCR, the difference did not reach statistical significance, likely due to the small sample size. A recent meta-analysis of *Mucorales* PCR showed a sensitivity of 86.4% (95% CI 78.9%–91.5%) in tissue samples, which suggests that it is a reliable tool when mucormycosis is suspected (10). The EORTC-MSGERC panel favors the use of panfungal PCR over fungal-specific PCRs (12). While panfungal PCR can detect a broader range of fungal pathogens (i.e., not included in the specific PCR panel), our opinion is that fungal-specific PCRs should be performed concomitantly with panfungal PCR in case of clinical suspicion and consistent histopathology findings. This approach may avoid delay in diagnosis and improve sensitivity for the detection of the most frequent fungal pathogens (included in the panel) (47).

A major concern about panfungal PCR remains its specificity when testing histopathology-negative samples. Specificity of panfungal PCR could be assessed for FFPE samples only and on a limited data set. Our results indicate good specificity (93.5%). Of note, false positive results should be interpreted cautiously, as the negative controls may have included patients with suspected IFD, for which standard methods (i.e., histopathology or culture) failed to identify the pathogen. The clinical utility of panfungal PCR in histopathology-negative tissue samples for patients with suspected IFD is still debated. The EORTC-MSGERC panel stated that PCR is only indicated when fungal elements have been detected by histopathology (4, 12). Indeed, results of panfungal PCR might be difficult to interpret in histopathology-negative samples because of possible environmental contamination. This approach is supported by one study showing high negative predictive value for IFD when fungal elements are not seen at histopathology (56). However, IFD is often associated with low fungal burden in abundant necrotic tissue, where PCR would be able to detect low amounts of fungi despite negative histopathology results. Few studies provided results of panfungal PCR on histopathology-negative tissue samples in patients with probable or possible IFD. Ala-Houhala et al. found relevant PCR results in 3/9 patients with a probable IFD (24). Babouee et al. reported positive findings in 3/8 patients with possible IFD (25). The actual relevance and added value of panfungal PCR on fresh (non-fixed) tissue samples for cases with high suspicion of IFD and negative histopathology of a companion sample should be prospectively investigated.

Fungal PCRs were only recently implemented in the revised EORTC-MSGERC definitions following improvements in standardization of DNA extraction methods and analytical procedures (4, 6, 57). Interlaboratory evaluations to assess protocol reproducibility have been conducted for *Aspergillus*, *Pneumocystis*, and *Mucorales* PCRs (8, 9, 57–59). However, such studies are still lacking for panfungal PCR, where the provision of contrived sample panels is complicated. Moreover, most laboratories use in-house protocols in the absence of commercial kits for fungal diagnostics. Not surprisingly, the quality assessment showed applicability concerns related to differences in laboratory methods, and our results display substantial heterogeneity (mainly for FFPE). While our statistical model accounts for intra- and inter-study heterogeneity, the lack of detectable modifiers in sub-analyses may be attributed to unmeasured factors of heterogeneity (e.g., DNA quality, fungal burden, human/fungal DNA ratio, sequencing performance). The type of PCR target is crucial for sensitivity and accuracy of genus/species identification. However, the low sample size and the heterogeneity of PCR targets precluded robust comparative analyses. Moreover, other important information that can impact PCR performance, such as the amount of tissue, the extraction methods, the formalin fixation time, and the quality control procedures (positive/negative and inhibition controls), was lacking in many studies or displayed too much heterogeneity for sub-analyses. This observation highlights the need for implementation of standardized procedures for panfungal PCR in tissue samples.

In conclusion, this study shows that panfungal PCR provides adequate sensitivity (75%) and good specificity (93.5%) when testing histopathology-positive FFPE tissue samples, confirming its role for genus/species level identification. Importantly, sensitivity was even higher (86.5%) in histopathology-positive non-fixed tissue samples, where culture sensitivity was low (approximately 50%). These results outline the importance of performing panfungal PCR on both sample types when histopathology shows fungal elements. The use of fungal-specific PCRs may enhance sensitivity further and should be considered in addition to panfungal PCR, according to the level of clinical suspicion. The potential utility of performing panfungal PCR on fresh tissue samples in case of high IFD suspicion despite negative histopathology samples should be assessed in prospective cohorts. Whether new panfungal PCR platforms or commercial kits can improve performance should be assessed in the future. Interlaboratory assays to compare different PCR targets, assess reproducibility, and optimize analytical protocols are therefore needed.

## SUPPLEMENTAL MATERIAL

Supplemental material jcm.00721-26-s0001.docx Supplemental methods and results.

ASM does not own the copyrights to Supplemental Material that may be linked to, or accessed through, an article. The authors have granted ASM a non-exclusive, world-wide license to publish the Supplemental Material files. Please contact the corresponding author directly for reuse.
