# Supplementary material for: Diagnostic performance of panfungal PCR on tissue specimens for the diagnosis of invasive fungal diseases: a systematic review and meta-analysis of the Fungal PCR Initiative (FPCRI)
Source: J Clin Microbiol. 2026 Aug 21;64(9):e00721-26. doi: 10.1128/jcm.00721-26 (PMC13556222; doi:10.1128/jcm.00721-26)
Supplement: Supplementary file 1 [file jcm.00721-26-s0001.docx]

**SUPPLEMENTARY MATERIAL**

**Diagnostic performance of panfungal PCR on tissue specimens for the diagnosis of invasive fungal diseases: a systematic review and meta-analysis of the Fungal PCR Initiative (FPCRI)**

Paraskevas FILIPPIDIS, Céline EL KHOURY, Mario CRUCIANI, Alexandre ALANIO, Rosemary A BARNES, Ralf BIALEK, Maria Jose BUITRAGO, J. Peter DONNELLY, Raphael GROLIMUND, Ferry HAGEN, Catriona HALLIDAY, Elizabeth JOHNSON, Martina LENGEROVA, Jurgen LOEFFLER, Willem JG MELCHERS, Carlo MENGOLI, Lily NOVAK-FRAZER, Onya OPOTA, Melinda PAHOLCSEK, Thomas F PATTERSON, Riina RAUTEMAA-RICHARDSON, Volker RICKERTS, Markus RUHNKE, Lena TSCHIDERER, Alexia CAVIN-TROMBERT, Birgit WILLINGER, P. Lewis WHITE, Frederic LAMOTH

**Supplementary Material S1. Preferred Reporting Items for Systematic Reviews and Meta-Analysis for Diagnostic Test Accuracy (PRISMA-DTA) Checklist Item**...………. Pages 2 – 4

**Supplementary Material S2. Database Search Strategies**...………………………. Pages 5 – 7

**Supplementary Material S3. Risk of bias and applicability concerns per-study graph** …………………………………………………………….………………………………………. Page 8

**Supplementary Material S4. Meta-regression analysis across potential sources of heterogeneity** ……………………………………………………………………………Pages 9 – 10

**Supplementary Material S5. Specificity of panfungal PCR for fungal detection / identification in formalin-fixed paraffin-embedded (FFPE) samples** ……………… Page 11

**Supplementary Material S6. Comparative analysis of sensitivity between *Mucorales*-specific and panfungal PCR for detection/identification of Mucorales in histopathology samples** ………………………………………………………………………………………. Page 12

**Supplementary Material S1. Preferred Reporting Items for Systematic Reviews and Meta-Analysis for Diagnostic Test Accuracy (PRISMA-DTA) Checklist Item**

| **Section/topic** | **#** | **PRISMA-DTA Checklist Item** | **Reported on page #** |
| --- | --- | --- | --- |
| **TITLE / ABSTRACT** | | |  |
| Title | 1 | Identify the report as a systematic review (+/- meta-analysis) of diagnostic test accuracy (DTA) studies. | 1 |
| Abstract | 2 | Abstract: See PRISMA-DTA for abstracts. | 3 |
| **INTRODUCTION** | | |  |
| Rationale | 3 | Describe the rationale for the review in the context of what is already known. | 5 |
| Clinical role of index test | D1 | State the scientific and clinical background, including the intended use and clinical role of the index test, and if applicable, the rationale for minimally acceptable test accuracy (or minimum difference in accuracy for comparative design). | 5 |
| Objectives | 4 | Provide an explicit statement of question(s) being addressed in terms of participants, index test(s), and target condition(s). | 5 |
| **METHODS** | | |  |
| Protocol and registration | 5 | Indicate if a review protocol exists, if and where it can be accessed (e.g., Web address), and, if available, provide registration information including registration number. | 6 |
| Eligibility criteria | 6 | Specify study characteristics (participants, setting, index test(s), reference standard(s), target condition(s), and study design) and report characteristics (e.g., years considered, language, publication status) used as criteria for eligibility, giving rationale. | 7 |
| Information sources | 7 | Describe all information sources (e.g., databases with dates of coverage, contact with study authors to identify additional studies) in the search and date last searched. | 6 |
| Search | 8 | Present full search strategies for all electronic databases and other sources searched, including any limits used, such that they could be repeated. | 6 |
| Study selection | 9 | State the process for selecting studies (i.e., screening, eligibility, included in systematic review, and, if applicable, included in the meta-analysis). | 7 |
| Data collection process | 10 | Describe method of data extraction from reports (e.g., piloted forms, independently, in duplicate) and any processes for obtaining and confirming data from investigators. | 7 |
| Definitions for data extraction | 11 | Provide definitions used in data extraction and classifications of target condition(s), index test(s), reference standard(s) and other characteristics (e.g. study design, clinical setting). | 7 |
| Risk of bias and applicability | 12 | Describe methods used for assessing risk of bias in individual studies and concerns regarding the applicability to the review question. | 8 |
| Diagnostic accuracy measures | 13 | State the principal diagnostic accuracy measure(s) reported (e.g. sensitivity, specificity) and state the unit of assessment (e.g. per-patient, per-lesion). | 8-9 |
| Synthesis of results | 14 | Describe methods of handling data, combining results of studies and describing variability between studies. This could include, but is not limited to: a) handling of multiple definitions of target condition. b) handling of multiple thresholds of test positivity, c) handling multiple index test readers, d) handling of indeterminate test results, e) grouping and comparing tests, f) handling of different reference standards | 8-9 |

| **Section/topic** | **#** | **PRISMA-DTA Checklist Item** | **Reported on page #** |
| --- | --- | --- | --- |
| Meta-analysis | D2 | Report the statistical methods used for meta-analyses, if performed. | 9 |
| Additional analyses | 16 | Describe methods of additional analyses (e.g., sensitivity or subgroup analyses, meta-regression), if done, indicating which were pre-specified. | 8-9 |
| **RESULTS** | | |  |
| Study selection | 17 | Provide numbers of studies screened, assessed for eligibility, included in the review (and included in meta-analysis, if applicable) with reasons for exclusions at each stage, ideally with a flow diagram. | 10 |
| Study characteristics | 18 | For each included study provide citations and present key characteristics including a) participant characteristics (presentation, prior testing), b) clinical setting, c) study design, d) target condition definition, e) index test, f) reference standard, g) sample size, h) funding sources | 10 |
| Risk of bias and applicability | 19 | Present evaluation of risk of bias and concerns regarding applicability for each study. | 10 |
| Results of individual studies | 20 | For each analysis in each study (e.g. unique combination of index test, reference standard, and positivity threshold) report 2x2 data (TP, FP, FN, TN) with estimates of diagnostic accuracy and confidence intervals, ideally with a forest or receiver operator characteristic (ROC) plot. | 10-11 |
| Synthesis of results | 21 | Describe test accuracy, including variability; if meta-analysis was done, include results and confidence intervals. | 10-11 |
| Additional analysis | 23 | Give results of additional analyses, if done (e.g., sensitivity or subgroup analyses, meta-regression; analysis of index test: failure rates, proportion of inconclusive results, adverse events). | 11 |
| **DISCUSSION** | | |  |
| Summary of evidence | 24 | Summarize the main findings including the strength of evidence. | 12 |
| Limitations | 25 | Discuss limitations from included studies (e.g. risk of bias and concerns regarding applicability) and from the review process (e.g. incomplete retrieval of identified research). | 13-14 |
| Conclusions | 26 | Provide a general interpretation of the results in the context of other evidence. Discuss implications for future research and clinical practice (e.g. the intended use and clinical role of the index test). | 14-15 |
| **FUNDING** | | |  |
| Funding | 27 | For the systematic review, describe the sources of funding and other support and the role of the funders. | N/A |

*Adapted From:* McInnes MDF, Moher D, Thombs BD, McGrath TA, Bossuyt PM, The PRISMA-DTA Group (2018). Preferred Reporting Items for a Systematic Review and Meta-analysis of Diagnostic Test Accuracy Studies: The PRISMA-DTA Statement. JAMA. 2018 Jan 23;319(4):388-396. doi: 10.1001/jama.2017.19163

**Supplementary Material S2. Database Search Strategies**

## Embase.com

conference abstracts, conference reviews, and animal studies excluded

**2118 results on May 27, 2025**

('systemic mycosis'/exp OR 'lung mycosis'/de OR 'Pneumocystis pneumonia'/de OR 'pulmonary candidiasis'/de OR 'pulmonary cryptococcosis'/de OR 'central nervous system fungal infection'/exp OR 'dimorphic fungal infection'/exp OR 'fungal sinusitis'/de OR 'zygomycosis'/de OR 'mucormycosis'/de OR 'aspergillosis'/exp OR 'fungal endocarditis'/exp OR 'fungal eye infection'/de OR 'Candida endophthalmitis'/de OR 'fungal endophthalmitis'/de OR 'ocular histoplasmosis'/de OR 'candidiasis'/de OR (IFI OR IFD OR fungal OR fungus OR fungi OR ((systemic OR invasive OR disseminat*) NEAR/3 mycos*) OR aspergillosis OR aspergillus OR mucor* OR rhizopus OR fusarium OR cryptococcus OR paracryptococcus OR histoplasma OR histoplasmosis OR coccidioidomycosis OR cryptococcosis OR candidiasis OR rhinosporidiosis OR zygomycete* OR absidia OR apophysomyces OR cunninghamella OR saksenaea):ab,ti,kw) AND ('polymerase chain reaction'/exp OR 'DNA extraction'/de OR 'RNA extraction'/de OR 'sequence analysis'/de OR 'DNA sequencing'/de OR 'RNA sequencing'/de OR 'fungal DNA'/de OR 'fungal RNA'/de OR (PCR* OR qPCR* OR "polymerase chain reaction*" OR "nucleic acid amplification*" OR ((fungal OR fungus OR fungi OR sequenc*) NEAR/8 (DNA OR RNA)) OR (molecular NEAR/3 diagnos*) OR ((fungal OR fungus OR fungi OR molecular OR sequenc* OR DNA OR RNA) NEAR/3 (typing OR assay* OR analys* OR extract*)) OR ((molecular OR sequenc*) NEAR/3 (method OR methods))):ab,ti,kw) AND ('biopsy'/exp OR 'ascites fluid'/de OR 'pleura fluid'/de OR 'cerebrospinal fluid'/de OR 'synovial fluid'/de OR 'parenchyma'/exp OR 'tissue by organ'/exp OR 'tissue embedding'/exp OR (biops* OR ((tissue* OR sampl* OR fluid OR fluids OR specimen* OR site* OR body OR bodies) NEAR/3 (sterile OR deep OR fresh OR native)) OR paraffin* OR formaldehyde* OR formalin* OR FFP OR FFPE):ab,ti,kw) AND ('fungal cell culture'/de OR 'immunohistochemistry'/exp OR 'histopathology'/de OR 'mycology'/de OR 'fungal examination'/exp OR 'histology'/de OR 'gold standard'/de OR 'diagnostic test accuracy study'/exp OR 'comparative study'/exp OR 'diagnostic accuracy'/de OR 'measurement accuracy'/exp OR 'sensitivity and specificity'/de OR 'reference value'/exp OR 'diagnostic error'/exp OR (microscopy OR microscopic OR culture* OR histopatho* OR cytopatho* OR microbiol* OR immunohisto* OR histolog* OR histomorpholog* OR mycology OR mycological* OR "European Organization for Research and Treatment of Cancer" OR EORTC* OR ((gold* OR standard* OR usual OR referenc* OR traditional*) NEAR/8 (method* OR criteri*)) OR (traditional* NEXT/1 (detection OR diagnos*)) OR ((gold* OR referenc*) NEXT/1 (standard* OR test*)) OR specificit* OR sensitivit* OR ((diagnos* OR test OR tests) NEAR/3 (accura* OR reliab* OR validat*)) OR ((positive OR negative) NEXT/1 (value* OR predict*)) OR (false NEXT/1 (positive OR negative))):ab,ti,kw) NOT ('conference abstract'/it OR 'conference review'/it) NOT ([animals]/lim NOT [humans]/lim)

## Medline Ovid ALL

1946 to May 26, 2025

**971 results on May 27, 2025**

(Mycoses/ OR exp * Mycoses/ OR exp Invasive Fungal Infections/ OR exp Lung Diseases, Fungal/ OR exp Central Nervous System Fungal Infections/ OR Coccidioidomycosis/ OR Histoplasmosis/ OR Lobomycosis/ OR Sporotrichosis/ OR Allergic Fungal Sinusitis/ OR exp Zygomycosis/ OR exp Aspergillosis/ OR ((Endocarditis/ OR Endocarditis, Bacterial/ OR Endocarditis, Subacute Bacterial/) AND Fungi/) OR exp Eye Infections, Fungal/ OR (exp Endophthalmitis/ AND (Candida/ OR Fungi/)) OR Candidiasis/ OR (IFI OR IFD OR fungal OR fungus OR fungi OR ((systemic OR invasive OR disseminat*) ADJ3 mycos*) OR aspergillosis OR aspergillus OR mucor* OR rhizopus OR fusarium OR cryptococcus OR paracryptococcus OR histoplasma OR histoplasmosis OR coccidioidomycosis OR cryptococcosis OR candidiasis OR rhinosporidiosis OR zygomycete* OR absidia OR apophysomyces OR cunninghamella OR saksenaea).ab,ti,kf.) AND (exp Polymerase Chain Reaction/ OR Sequence Analysis/ OR Sequence Analysis, DNA/ OR Sequence Analysis, RNA/ OR DNA, Fungal/ OR RNA, Fungal/ OR (PCR* OR qPCR* OR "polymerase chain reaction*" OR "nucleic acid amplification*" OR ((fungal OR fungus OR fungi OR sequenc*) ADJ8 (DNA OR RNA)) OR (molecular ADJ3 diagnos*) OR ((fungal OR fungus OR fungi OR molecular OR sequenc* OR DNA OR RNA) ADJ3 (typing OR assay* OR analys* OR extract*)) OR ((molecular OR sequenc*) ADJ3 (method OR methods))).ab,ti,kf.) AND (exp Biopsy/ OR Ascitic Fluid/ OR Pleural Cavity/ OR Cerebrospinal Fluid/ OR Synovial Fluid/ OR Parenchymal Tissue/ OR exp Tissue Embedding/ OR (biops* OR ((tissue* OR sampl* OR fluid OR fluids OR specimen* OR site* OR body OR bodies) ADJ3 (sterile OR deep OR fresh OR native)) OR paraffin* OR formaldehyde* OR formalin* OR FFP OR FFPE).ab,ti,kf.) AND (exp Cell Culture Techniques/ OR exp Immunohistochemistry/ OR Histology/ OR Mycology/ OR Diagnostic Tests, Routine/ OR Comparative Study/ OR exp "Sensitivity and Specificity"/ OR Reference Standards/ OR Diagnostic Reference Levels/ OR Reference Values/ OR exp Diagnostic Errors/ OR (microscopy OR microscopic OR culture* OR histopatho* OR cytopatho* OR microbiol* OR immunohisto* OR histolog* OR histomorpholog* OR mycology OR mycological* OR "European Organization for Research and Treatment of Cancer" OR EORTC* OR ((gold* OR standard* OR usual OR referenc* OR traditional*) ADJ8 (method* OR criteri*)) OR (traditional* ADJ (detection OR diagnos*)) OR ((gold* OR referenc*) ADJ (standard* OR test*)) OR specificit* OR sensitivit* OR ((diagnos* OR test OR tests) ADJ3 (accura* OR reliab* OR validat*)) OR ((positive OR negative) ADJ (value* OR predict*)) OR (false ADJ (positive OR negative))).ab,ti,kf.) NOT (exp animals/ not humans.sh.)

## Cochrane Library Wiley

Cochrane Database of Systematic Reviews Issue 5 of 12, May 2025

Cochrane Central Register of Controlled Trials Issue 5 of 12, May 2025

**22 results on May 27, 2025**

(IFI OR IFD OR fungal OR fungus OR fungi OR ((systemic OR invasive OR disseminat*) NEAR/3 mycos*) OR aspergillosis OR aspergillus OR mucor* OR rhizopus OR fusarium OR cryptococcus OR paracryptococcus OR histoplasma OR histoplasmosis OR coccidioidomycosis OR cryptococcosis OR candidiasis OR rhinosporidiosis OR zygomycete* OR absidia OR apophysomyces OR cunninghamella OR saksenaea):ab,ti,kw AND (PCR* OR qPCR* OR ("polymerase chain" NEXT reaction*) OR ("nucleic acid" NEXT amplification*) OR ((fungal OR fungus OR fungi OR sequenc*) NEAR/8 (DNA OR RNA)) OR (molecular NEAR/3 diagnos*) OR ((fungal OR fungus OR fungi OR molecular OR sequenc* OR DNA OR RNA) NEAR/3 (typing OR assay* OR analys* OR extract*)) OR ((molecular OR sequenc*) NEAR/3 (method OR methods))):ab,ti,kw AND (biops* OR ((tissue* OR sampl* OR fluid OR fluids OR specimen* OR site* OR body OR bodies) NEAR/3 (sterile OR deep OR fresh OR native)) OR paraffin* OR formaldehyde* OR formalin* OR FFP OR FFPE):ab,ti,kw AND (microscopy OR microscopic OR culture* OR histopatho* OR cytopatho* OR microbiol* OR immunohisto* OR histolog* OR histomorpholog* OR mycology OR mycological* OR "European Organization for Research and Treatment of Cancer" OR EORTC* OR ((gold* OR standard* OR usual OR referenc* OR traditional*) NEAR/8 (method* OR criteri*)) OR (traditional* NEXT (detection OR diagnos*)) OR ((gold OR referenc*) NEXT (standard* OR test*)) OR specificit* OR sensitivit* OR ((diagnos* OR test OR tests) NEAR/3 (accura* OR reliab* OR validat*)) OR ((positive OR negative) NEXT (value* OR predict*)) OR (false NEXT (positive OR negative))):ab,ti,kw

## Web of Science – Core collection

Advanced Search > Exact search

**886 results on May 27, 2025**

TS=((IFI OR IFD OR fungal OR fungus OR fungi OR ((systemic OR invasive OR disseminat*) NEAR/2 mycos*) OR aspergillosis OR aspergillus OR mucor* OR rhizopus OR fusarium OR cryptococcus OR paracryptococcus OR histoplasma OR histoplasmosis OR coccidioidomycosis OR cryptococcosis OR candidiasis OR rhinosporidiosis OR zygomycete* OR absidia OR apophysomyces OR cunninghamella OR saksenaea) AND (PCR* OR qPCR* OR "polymerase chain reaction*" OR "nucleic acid amplification*" OR ((fungal OR fungus OR fungi OR sequenc*) NEAR/7 (DNA OR RNA)) OR (molecular NEAR/2 diagnos*) OR ((fungal OR fungus OR fungi OR molecular OR sequenc* OR DNA OR RNA) NEAR/2 (typing OR assay* OR analys* OR extract*)) OR ((molecular OR sequenc*) NEAR/2 (method OR methods))) AND (biops* OR ((tissue* OR sampl* OR fluid OR fluids OR specimen* OR site* OR body OR bodies) NEAR/2 (sterile OR deep OR fresh OR native)) OR paraffin* OR formaldehyde* OR formalin* OR FFP OR FFPE) AND (microscopy OR microscopic OR culture* OR histopatho* OR cytopatho* OR microbiol* OR immunohisto* OR histolog* OR histomorpholog* OR mycology OR mycological* OR "European Organization for Research and Treatment of Cancer" OR EORTC* OR ((gold* OR standard* OR usual OR referenc* OR traditional*) NEAR/7 (method* OR criteri*)) OR (traditional* NEAR/0 (detection OR diagnos*)) OR ((gold* OR referenc*) NEAR/0 (standard* OR test*)) OR specificit* OR sensitivit* OR ((diagnos* OR test OR tests) NEAR/2 (accura* OR reliab* OR validat*)) OR ((positive OR negative) NEAR/0 (value* OR predict*)) OR (false NEAR/0 (positive OR negative))))

## Google Scholar

Research without the "sterile" concept
First 500 references exported via Harzing's Publish or Perish (Windows GUI Edition) 8.6.4198.8332 – No patents, no citations
**500 results on May 27, 2025**

fungal|fungus|fungi|mycosis systemic|invasive|disseminated PCR|qPCR tissue|biopsy microscopic|culture|histopathology|microbiology|"gold standard"|"standard method"|"test accuracy"|sensitivity|specificity|"positive predictive"|"negative predictive"

**Supplementary Material S3. Risk of bias and applicability concerns per-study graph.**

Risk of bias and applicability concerns summary: individual assessment of methodological quality of the included studies using the Quality Assessment of Diagnostic Accuracy Studies (QUADAS-2) tool.

**Supplementary Material S4. Meta-regression analysis across potential sources of heterogeneity.**

|  | FFPE samples  Sensitivity (95% CI)  [N studies / N cases] | *P-value* | Non-fixed samples  Sensitivity (95% CI)  [N studies / N cases] | *P-value* |
| --- | --- | --- | --- | --- |
| Tissue origin |  | 0.068 |  | 0.557 |
| Biopsy | 0.65 (0.37, 0.86)  [4 / 251] |  | 0.80 (0.59, 0.92)  [6 / 101] |  |
| Autopsy | 0.18 (0.02, 0.70)  [1 / 49] |  | na |  |
| Biopsy and Autopsy | 0.89 (0.67, 0.97)  [4 / 227] |  | 0.85 (0.38, 0.98)  [1 / 12] |  |
| Missing | 0.76 (0.57, 0.88)  [9 / 325] |  | 0.89 (0.77, 0.96)  [6 / 94] |  |
| Tissue type |  | 0.470 |  | 0.564 |
| Lung (majority)^¶^ | 0.67 (0.35, 0.88)  [5 / 203] |  | 0.90 (0.59, 0.98)  [2 / 25] |  |
| Non-lung (majority)^¶^ | 0.85 (0.62, 0.95)  [6 / 223] |  | 0.87 (0.74, 0.94)  [10 / 163] |  |
| Missing | 0.70 (0.45, 0.88)  [7 / 426] |  | 0.69 (0.23, 0.94)  [2 / 25] |  |
| PCR target* |  | 0.183 |  | 0.913 |
| 18S | na |  | 0.89 (0.53, 0.98)  [2 / 20] |  |
| 28S | 0.86 (0.57, 0.96)  [4 / 123] |  | 0.75 (0.26, 0.96)  [1 / 19] |  |
| ITS | 0.65 (0.47, 0.80)  [13 / 608] |  | 0.85 (0.71, 0.93)  [9 / 160] |  |
| ITS + 28S/18S | 0.88 (0.59, 0.97)†  [3 / 153] |  | 0.83 (0.43, 0.97)§  [2 / 24] |  |
| PCR protocol |  | 0.837 |  | 0.925 |
| Real-time | 0.78 (0.53, 0.92)  [7 / 293] |  | 0.88 (0.62, 0.96)  [4 / 55] |  |
| Conventional | 0.71 (0.45, 0.88)  [8 / 420] |  | 0.85 (0.68, 0.94)  [7 / 132] |  |
| Missing | 0.80 (0.39, 0.96)  [3 / 139] |  | 0.89 (0.53, 0.98)  [2 / 20] |  |
| Extraction controls |  | 0.057 |  |  |
| Used | 0.62 (0.41, 0.80)  [10 / 480] |  | na |  |
| Not used | 0.86 (0.68, 0.94)  [8 / 372] |  | na |  |

Subgroup analyses of panfungal PCR sensitivity according to tissue origin, PCR target, PCR protocol, and use of extraction controls. Sensitivity estimates are shown for FFPE and native tissue samples using histopathology as the reference standard. P-values correspond to subgroup comparisons derived from mixed-effects meta-regression within the random-effects generalized linear mixed model by including variables as fixed effects. Sensitivities are presented with 95% confidence intervals (CI); brackets indicate the number of included studies and total number of samples.

FFPE: formalin-fixed paraffin-embedded, *na*, insufficient data or not applicable.

^¶^ “Majority” refers to studies including >60% samples from lung or non-lung tissues, respectively.

* Two and one study examined two different PCR targets in FFPE and native samples, respectively; therefore, these studies are represented twice in the meta-regression analysis of PCR targets.

† All studies utilized a combined ITS + 28S target.

§ One study utilized a combined ITS + 28S target and one a combined ITS + 18S target.

**Supplementary Material S5. Specificity of panfungal PCR for fungal detection / identification in formalin-fixed paraffin-embedded (FFPE) samples.**

Forest plots of specificity of panfungal PCR in formalin-fixed paraffin-embedded (FFPE) compared to histopathology (reference). Specificity estimates are presented with 95% confidence intervals. Pooled estimates were obtained using a random-effects model. Study heterogeneity is represented by the I^2^ statistic.

**Supplementary Material S6. Comparative analysis of sensitivity between *Mucorales*-specific and panfungal PCR for detection/identification of Mucorales in histopathology samples.**

Forest plot comparing sensitivity of *Mucorales*-specific PCR (top panel, MUC) and panfungal PCR (bottom panel, PANF) in native tissue samples from studies examining both assays. Sensitivity estimates are presented with 95% confidence intervals. Pooled estimates were obtained using a random-effects model. Study heterogeneity is represented by the I^2^ statistic.
